# Supplementary material for: Eating for honour: A cultural-ecological analysis of food behaviours among adolescent girls in the southern plains of Nepal
Source: PLoS One. 2023 Aug 18;18(8):e0290405. doi: 10.1371/journal.pone.0290405 (PMC10437861; doi:10.1371/journal.pone.0290405)
Supplement: Attachment — Submitted filename: GirlsEatRevResv1.docx [file pone.0290405.s002.docx]

We thank the reviewers for their comments and have responded to their concerns below.

Reviewer #1:

The introduction needs more focused on theoretical approach in a cultural setting. The authors may consider health systems studies to elaborate more particularly the role of diet in a household and overall health outcomes.

*We use the theoretical framework of the cultural ecological model. The data were not collected in the setting of the health system, nor refer to the health system. We sought to understand eating in the cultural context of the home and community.* *We have however added to the introduction section to strengthen the linkages between girls’ health and nutrition.*

The methodology need justification of sample, if required in a tabular format.

*The paper is based on a secondary analysis of data collected for a previous study. The sampling criteria are discussed on page 7 line 149.*

The result section is very lengthy, hence, important finding like cultural practices have to be highlighted.

*We discuss our results in reference to the theoretical framework (Figure 1), which highlight several factors affecting eating behaviours. We have edited the results somewhat to make them more concise.*

Reviewer #2: Comments to the author
Thank you for the opportunity to review this work which provides useful insight into the factors that influence adolescent girls diet behaviours in Nepal. While the paper presents useful information on socio-cultural factors that should be targeted, I would recommend that the authors revise the aim of the paper to make it clearer/more concise, as well as to cut down and streamline the introduction (see below). I would also recommend that the manuscript be reviewed for language and flow, as the narrative doesn’t always flow and this can compromise the motivation behind the study and there are several typos, e.g., in line 48, ‘need’ should be replaced with ‘needs’

*We have proofread the article and made changes. We have also substantially revised the introduction section.*

More detailed feedback is provided below.
General
Abstract
- Line 16-19: The opening lines of the abstract make a case for the importance of nutrition for adolescent girls and the influence of structural issues, but the authors could make a stronger link between the influence of socio-cultural and structural factors on adolescent girls dietary behaviours

*We have added to the abstract as suggested.*

- I recommend specifying the age range included in the study within the abstract

*We have added the age range*.
- Please specify the qualitative methodology used to collect the data; e.g. focus group discussions/ individual interviews/both, and the methods used to analyse the data in the abstract

*We have added the methods to the abstract and specified that we used the cultural -ecological framework to analyse the data.*

- Line 21: Please be more specific around what you refer to as ‘eating’ – was this the quality and/or quantity of food available/accessible to them?

*Our article focuses on eating, which refers to food consumption behaviour and all the factors that affect this. We have added this to the abstract to clarify.*

Introduction
- Line 38-41: While I agree that the points made around early marriage and pregnancy and the particular needs to ensure optimal nutrition for girls, I don’t think the links between early marriage, adolescent pregnancy and the nutritional status of adolescent girls, as well as the implications for themselves and their infants are clear. I suggest revising this to strengthen the linkages for the reader
- Line 42-43: the point ‘Dietary risks are the second leading contributor to Nepal’s burden of disease comes out of nowhere ate the end of the paragraph without links to the narrative being told. I suggest revising the paragraph for better flow

*We have revised this paragraph and added references.*

- Line 53-58: The aim presented for the study isn’t very clearly stated and I would argue that lines 56-58 provide information better suited to the discussion, as it relates more to the findings that the study presents/implications than the specific aim. As stated above, I suggest revising the aim to more clearly/concisely state what was done.

*We state the study aim on page 3 “to analyse what affects girls’ eating behaviour in rural plains Nepal.”*

*We have removed the lines suggested and adjusted the text on page 6 about the study aim.*

- The introductory section is extremely long and I find myself confused by why the aim of the study is presented, and then followed by additional background information on the links between culture and food/ food and Nepal and then aspects related to gender. I would recommend a complete revision of the introduction to more clearly and concisely present this background information, followed by clear reflection of the aim at the end.

*We have revised and shortened the introduction section, focusing on gender as a determinant of nutrition. We have moved information about the Nepali diet to the context section of the methods because our results about food and snacking needs this contextual information to be understood.*

Methods
- The data presented is now 10 years old and it is unclear of how representative these findings are to the current situation in Nepal. Have there been any shifts in cultural/social norms in the past decade and, if so, how would these influence the interpretation of the findings/ their applications to informing interventions. The authors should discuss this in the discussion section – while it is briefly touched on in the limitations, more insight into how the authors feel this reflects current practices is warranted.

*Our data represent a point in time, and due to the lack of research with this group in this setting, our findings are important to present. Although we find it difficult to speculate about exactly what the situation is for girls today without supporting evidence from girls or other research with girls, we have added a paragraph discussing the implications of some social changes, and note that gender inequalities persist in the plains areas of Nepal.*

*We have also added to the introductory section to demonstrate that the issue of gender is a* ***persistent*** *barrier to good nutrition of girls and women and then cited recent references (ie in the past few years), as well as a systematic review which contains some older references.*

- It would be useful to, alongside information on the number of FGDs, provide the number of participants per FGD.

*We have added information in the text about how many participants were in the FGDs (line 349).*
- The authors state that it was a preference not to use qualitative analysis software – it would be useful to understand the motivation behind this/to discuss whether this may have been a limitation in the discussion section. It would be useful to also understand more around the coding of the data – the methods state that coding was done using highlighter pens, but what was the basis of the coding and how was this informed. The way it is currently described, the approach seems a bit unstructured and it is unclear how the analysis was compared between researchers – did both complete the coding for all FGDs and this was compared? Or was coding for each FGD only some by one researcher and then the comparisons made only across groups. Also, how was the coding them brought together – e.g. was thematic analysis used to group themes/sub-themes? I think you need to be clearer on your analysis methods and the justification to provide more insight into the ‘robustness’ of the methodology, as well as more clearly present the data.

*Researchers did not have equal access to software, and we felt it was important to undertake collaborative analysis. Using the software may have prevented this collaborative approach. We have added description of how we did the analysis.*

Results
- The ‘themes’ presented in the results are quite overlapping (e.g. Physical and social environment of eating and social organisation) and it is not clear what has informed the grouping of data/themes. This also means that sub-themes become a bit repetitive. I would suggest revising the information into more clearly defined themes that, in combination, describe the overall factors influencing adolescent girls diets

*As stated in our methods section, we used the cultural ecological framework to group the data and themes. We have added description of what is considered under each component of the framework at the beginning of each section to remind the reader of the structure.*

Discussion
- While the discussion provides useful discussion of the findings around social/cultural norms in Nepal, the authors could strengthen the links between these norms and the impact of what and where adolescent girls eat and the implications of this.
- Perhaps it would be helpful for the authors to comment on how these findings might inform intervention development (e.g. intervention targets/modalities) and any remaining gaps that require explanation. In addition, it would be useful to understand the role of adolescent boys and men in these contexts and this may be worth greater emphasis.

*We have substantially edited our discussion section, adding in sections on the relevance of our findings in the current context, and the need to consider men and boys. In our discussion we have made several recommendations which are summarised in our edited conclusion section.*

We look forward to hearing from you.
